# Modulatory Effects of Polyphenols on Altered Leukocyte Functions in Thromboinflammation and Diabetes Mellitus

**DOI:** 10.3390/ijms27083585

**Published:** 2026-04-17

**Authors:** Dina Muharib, Xinyi Wu, Christine Boesch, Robert A. S. Ariëns, Julia S. Gauer

**Affiliations:** 1 Discovery and Translational Science Department, Leeds Institute of Cardiovascular and Metabolic Medicine, University of Leeds, Leeds LS2 9JT, UK; sdtc2649@leeds.ac.uk (D.M.); kdtf4577@leeds.ac.uk (X.W.); r.a.s.ariens@leeds.ac.uk (R.A.S.A.); 2 School of Food Science and Nutrition, Faculty of Environment, University of Leeds, Leeds LS2 9JT, UK; c.bosch@leeds.ac.uk

**Keywords:** diabetes mellitus, leukocytes, polyphenols, thromboinflammation

## Abstract

Diabetes mellitus (DM) is a chronic metabolic disorder characterized by persistent low-grade inflammation and a markedly increased risk of cardiovascular diseases (CVD). Leukocytes play an important role not only in host defense but are also increasingly recognized as key contributors to haemostasis and thromboinflammatory processes. In DM, chronic hyperglycaemia, oxidative stress and inflammation lead to leukocyte dysfunction, including enhanced cell activation, impaired mitochondrial function, and dysregulated interactions with platelets and endothelial cells. These alterations promote the thromboinflammatory state that contributes to vascular complications in DM. Thus, the modulation of oxidative stress and inflammation are important. Polyphenols are a class of plant secondary metabolites widely studied for their antioxidant and anti-inflammatory properties. This review comprehensively explores leukocyte dysfunction in DM, its contribution to thromboinflammation, and the mechanistic role of polyphenols in modulating these processes. The evidence presented suggests that polyphenols may contribute to the modulation of thromboinflammatory pathways. Further research in this area is required to enhance our understanding of thromboinflammation in DM and to translate these findings into effective adjunctive strategies, alongside standard pharmacological therapies to reduce CVD risk in individuals with DM.

## 1. Introduction

Diabetes mellitus (DM) is a group of metabolic disorders characterized by persistent hyperglycaemia defined as a fasting plasma glucose level ≥ 7.0 mmol/L or a random plasma glucose level ≥ 11.1 mmol/L in presence of symptoms or signs of DM or hemoglobin A1c (HbA1c) exceeding 48 mmol/mol [1]. In contrast, normal glucose regulation is characterized by fasting glucose levels of 4.0–5.4 mmol/L, random plasma glucose ≤ 7.7 mmol/L, and HbA1c ≤ 41 mmol/mol [2]. These abnormalities result from deficient insulin secretion (type 1), resistance to the action of insulin (type 2), or both [3]. The global prevalence of DM has increased dramatically in recent decades, particularly through an increase in type 2 DM (T2DM) driven by a global rise in obesity. In 2022, approximately 14% of adults aged 18 years and older were living with DM, a rise from 7% in 1990 [4]. This growing prevalence contributes to an increase in mortality and caused 3.4 million deaths in 2024, approximately one death every six seconds [5].

Cardiovascular morbidities are the leading cause of mortality in DM [6]. In addition to traditional cardiovascular risk factors, DM is recognized as a thromboinflammatory condition characterized by dysregulated haemostasis and chronic low-grade inflammation [7,8]. This condition combined with chronic hyperglycaemia promotes thromboinflammation and accelerates the progression of micro- and macrovascular complications [7,9].

Recent evidence has highlighted the role of leukocytes including neutrophils, monocytes and lymphocytes in thromboinflammation [10]. Polyphenols are naturally occurring plant secondary metabolites, with over 40,000 compounds identified to date. As well as playing an important role in plant defense, polyphenols have increasingly demonstrated antioxidant, anti-inflammatory and immunomodulatory properties in mammalian cells [11,12]. Emerging evidence indicates that polyphenols may modulate leukocyte bioenergetics, mitochondrial function and oxidation, therefore modulating thromboinflammation in DM [13,14,15]. Also, observational and interventional studies suggest that higher polyphenol intake is associated with improved glucose levels and a lower incidence of T2DM [16,17]. This review explores how altered leukocyte bioenergetics contribute to the development of thromboinflammation in DM and evaluates the potential role of polyphenols in modulating leukocyte function. To our knowledge, this is the first description of the role of leukocyte bioenergetics in thromboinflammatory mechanisms and the effects of polyphenols in the context of DM.

## 2. Materials and Methods

The literature search was conducted using PubMed and MEDLINE via Ovid. Only English language, full-text papers published up to December 2025 were considered. Search terms related to diabetes included “diabetes mellitus”, “T2DM”, “T1DM” and “hyperglycaemia”, which were combined with thromboinflammation terms including “thromboinflammation”, “thrombosis”, “haemostasis”, “inflammation”, “platelet activation”, “cytokines”, “oxidation” and “endothelial activation”. Leukocytes-related terms included “leukocytes”, “neutrophils”, “lymphocytes”, “monocytes” and “macrophages”, whereas polyphenols’ keywords included “polyphenols”, “flavonoids”, stilbenes”, lignans”, “phenolic acids”, “resveratrol”, “EGCG”, and “quercetin”. Human, animal and in vitro studies that investigated the effects of polyphenols on thromboinflammation in diabetes were eligible for inclusion.

## 3. Cardiovascular Disease in DM

CVD complications represent the primary cause of morbidity and mortality in DM. Data from the Swedish National Diabetes Register, with approximately five years follow up for individuals with and without T2DM, showed that CVD caused nearly two thirds of all deaths [6]. Coronary heart disease was the predominant cause, followed by myocardial infarction (MI), atrial fibrillation, heart failure and stroke. Among individuals with DM, the mortality risk from CVD was highest in younger people and in those with poor glycaemic control or renal complications [6].

DM increases the risk of CVD, with disease onset occurring up to fifteen years earlier than in individuals without DM [18]. The EpiDREAM cohort trial showed that each 1 mmol/L increase in fasting plasma glucose is associated with a 17% higher risk of CVD events or death in individuals with T2DM, impaired fasting glucose or impaired glucose tolerance [19]. Furthermore, in a population-based autopsy study, individuals with DM without a prior coronary artery disease (CAD) diagnosis exhibited a high prevalence of coronary atherosclerosis, comparable to that observed in individuals diagnosed with CAD but without DM [20]. Consistent with this elevated risk, individuals with T2DM and without a previous incidence of MI have a similar risk of developing a MI compared to individuals without DM and history of MI, with an incidence of approximately 20% over seven years [21].

Several mechanisms contribute to the increased risk of CVD in DM. DM is associated with a prothrombotic haemostatic profile, characterized by elevated fibrinogen levels, the increased activity of coagulation factor VII (FVII) and a higher concentration of plasminogen activator inhibitor-1 (PAI-1), which collectively enhance coagulation and impair fibrinolysis [8,22,23,24]. Individuals with T2DM tend to form denser plasma fibrin clots that are resistant to fibrinolysis, which is associated with increased cardiovascular mortality [25]. Chronic low-grade inflammation is a key contributing factor in CVD in individuals with DM [7]. Inflammation and oxidative stress are essential physiological defense mechanisms against pathogens and play important roles in tissue repair [26]. However, in DM these processes become dysregulated, leading to persistently elevated baseline proinflammatory cytokine levels such as tumor necrosis factor (TNF)-α, interleukin (IL)-1ß, IL-8 and C-reactive protein (CRP) [27,28]. This chronic inflammatory state is largely driven by adipose tissue stress and the activation of innate immune responses that impair insulin signaling [29].

## 4. Leukocyte Role in CVD

Leukocytes are now recognized as active regulators of haemostasis and thrombosis, through their release of coagulation and fibrinolytic factors, and they provide procoagulant cellular surfaces [30]. Leukocytes, particularly monocytes, can express tissue factor (TF) under inflammatory conditions and thus drive the activation of the extrinsic coagulation cascade [31]. Consequently, monocytes may support thrombin formation on their cell surface. Thrombin generation requires the activation of coagulation factors through enzymatic complexes, a process that is facilitated on monocyte membranes through the binding of coagulation factors and their cofactors, promoting the efficient assembly and activity of these enzymatic complexes [32,33]. Activated monocytes release microparticles (MPs), which express TF and P-selectin glycoprotein ligand-1 (PSGL-1), enabling MP interaction with P-selectin on activated platelets and the transfer of procoagulant activity [34,35]. Furthermore, monocytes release proinflammatory cytokines that activate platelets and endothelial cells, thus enhancing cellular adhesion and thromboinflammatory responses [36,37].

Neutrophils adhere to activated endothelial cells prior to platelets, primarily through interaction with intercellular adhesion molecule-1 [38]. Upon activation, neutrophils release reactive oxygen species (ROS) as part of their innate immune response, mainly through the activation of nicotinamide adenine dinucleotide phosphate (NADPH) oxidase [39]. While ROS help to eliminate pathogens, they are involved in other processes, such as enhanced platelet activation, TF expression on monocytes, and the initiation of neutrophil extracellular trap (NET) formation, a process known as NETosis [40,41,42]. NET formation (NETosis) is promoted by the translocation of neutrophil elastase and myeloperoxidase to the nucleus, where neutrophil elastase cleaves histones, leading to chromatin decondensation, while myeloperoxidase further facilitates this process, resulting in NET release [43]. NETs are web-like structures that contain a matrix of deoxyribonucleic acid (DNA), histones and granule proteins, often referred as NETome, forming a scaffold that binds other cells, such as platelets and erythrocytes, thereby enhancing platelet activation, adhesion, aggregation and promoting thrombosis [44]. Histones in NETs promote thrombin generation via enhancing prothrombinase activity [45]. Histones promote procoagulant phenotypes in erythrocytes by inducing phosphatidylserine exposure on their surface, which facilitates the assembly of prothrombinase complexes, enhancing thrombin generation and promoting fibrin deposition [46]. In addition, neutrophils produce proinflammatory cytokines, which induce neutrophil activation and NETs’ formation [47]. Thus leukocytes, including neutrophils, lymphocytes and monocytes, have emerged as key contributors to thromboinflammation in DM. Elevated leukocyte counts were associated with increased CVD mortality, with neutrophil counts showing the strongest association [48]. Furthermore, neutrophil counts to the highest normal range (6 to 7 × 10^9^/L) were associated with the development of heart failure, peripheral arterial disease, unheralded coronary death and nonfatal MI during a four-year follow up period compared to the lowest normal range (2 to 3 × 10^9^/L) [49]. An elevation in the systemic immune inflammation index, which is a biomarker derived from platelet, neutrophil and lymphocyte counts, is also associated with increased prevalence of stroke [50]. Together, these observations suggest leukocytes contribute to the CVD risk in individuals with DM.

## 5. Altered Leukocyte Function in DM

**Figure 1:**
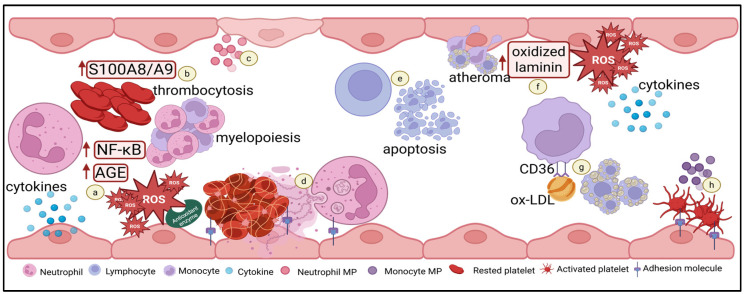
Leukocyte dysfunction in DM contributes to increased thrombosis risk. Chronic low-grade inflammation and hyperglycaemia in DM promote the activation of leukocytes, leading to functional alterations. In neutrophils, the following occurs: (**a**) upregulation of the advanced glycation end products (AGEs) formation, which activates signal transduction pathways, leading to excessive ROS production; increased ROS production, together with reduced antioxidant enzymes, leads to NF-κB activation, which enhances proinflammatory cytokines release; (**b**) increased S100A8/A9 production contributes to elevated cytokine levels and increased in platelet and leukocyte counts; (**c**) the release of neutrophil microparticles (MPs) expressing adhesion molecules and myeloperoxidase, which are related to changes in endothelial cell volume and loss of membrane integrity; and (**d**) this oxidative stress promotes NETosis, contributing to platelet activation, cell aggregation and clot formation. In lymphocytes, (**e**) impaired proliferation and dysregulated apoptosis occur, resulting in reduced circulating lymphocyte counts. In monocytes, the following occurs: (**f**) elevated ROS production and cytokine secretion, enhanced laminin oxidization and increased adhesion to laminin, which represents an early step in atheroma formation; (**g**) upregulated CD36 expression, which increased Ox-LDL uptake and foam cell formation; and (**h**) increased monocyte-derived MPs levels enhance platelet activation, as reflected by increased P-selectin and soluble E-selectin levels. Created in BioRender. Muharib, D. (2026) https://BioRender.com/kn4bwsv (accessed on 3 February 2026).

Leukocytes are the main cellular components of immune and inflammatory systems. They originate from nucleated precursor cells that differentiate and mature within the bone marrow into granulocytes: predominantly neutrophils, as well as eosinophils, and basophils, along with lymphocytes, and monocytes [51,52]. Figure 1 summarizes the contributions of altered leukocyte function towards DM discussed in the sections below.

### 5.1. Neutrophils

Neutrophils are the most abundant type of leukocytes, representing 50% to 70% of circulating leukocytes [53]. Initially, neutrophils were recognized for their role in the innate immune response against pathogens; however, growing evidence shows that neutrophils also display procoagulant and prothrombotic effect by releasing cytokines and inflammatory mediators [54].

DM impact several neutrophil functions, including degranulation, ROS production, proinflammatory cytokine release, and NETosis [27,55,56,57]. Hyperglycaemia drives the formation of advanced glycation end products (AGEs), activating signal transduction pathways leading to excessive ROS production. Elevated AGE levels are associated with the development of coronary heart disease in T2DM [58]. ROS production is associated with an increase in cytokine and chemokine synthesis via activating nuclear factor–κB (NF-κB). Additionally, the elevation in proinflammatory mediator levels increases ROS synthesis, thus inducing NF-κB activation [59]. Furthermore, neutrophil-derived S100 calcium-binding proteins A8/A9 (S100A8/A9) stimulate IL-6 production, promoting hepatic thrombopoietin synthesis and reticulated thrombocytosis [60]. Plasma S100A8/A9 levels are increased in type 1 DM (T1DM) and correlated with leukocyte counts and coronary artery disease [61]. Hyperglycaemia also promotes the release of neutrophil MPs [62]. Neutrophil MPs are membrane vesicles released upon activation such as by ROS production or apoptosis [62]. They express adhesion molecules such as CD62L and CD66b and myeloperoxidase (MPO), which are related to changes in endothelial cell volume and a loss of membrane integrity [63]. This inflammatory state contributes to NETosis, which can interact with the fibrin network generated through blood coagulation [64]. In plasma isolated from individuals with DM, the clot density was increased in the presence of NETs, and the fibrinolysis rate was slower after adjustment for fibrinogen levels [64]. These changes may contribute to the increase in arterial thrombosis in DM. Neutrophils isolated from individuals with DM showed a 4-fold increase in peptidyl arginine deiminase 4 (PAD4) protein expression compared to the healthy controls, an enzyme essential for initiating NETs formation through chromatin decondensation [65,66]. A study using samples from individuals with T2DM showed elevated plasma levels of NETosis markers, such as elastase, mono-oligonucleosomes and double-stranded DNA (dsDNA), which are positively correlated with CVD [67]. The increase in circulating NETs markers was correlated with prothrombotic state and hypofibrinolysis in this study. These markers were also significantly elevated in individuals with T2DM and MI, who also had prolonged clot lysis time (>100 min) associated with a 4.7-fold higher risk of concomitant CVD [68].

Conversely, NETosis levels and NETs markers were similar between T1DM and healthy controls [69]. However, the NETome differed in response to phorbol 12-myristate 13-acetate (PMA) and ionomycin stimulation. Neutrophils isolated from individuals with T1DM showed an upregulation of proteins involved in gluconeogenesis and glucose metabolism, while the innate immune response proteins were significantly reduced in the healthy control compared to T1DM. All in all, the aforementioned studies suggest that neutrophils induced changes in metabolic proteins to prevent bioenergetic impairment [69]. Alterations in neutrophil bioenergetics, including impaired oxygen consumption and mitochondrial complex I activity, have been shown to increase ROS levels, decrease GSH/GSSG ratio and reduce the mitochondrial membrane potential, which exacerbates thrombo-inflammatory responses [70].

### 5.2. Lymphocytes

Lymphocytes are peripheral blood mononuclear cells (PBMCs) that constitute approximately 25 to 40% of circulating leukocytes, include T-cells, B-cells, and natural killer cells [71]. DM alters lymphocyte metabolism, leading to functional impairments [72]. Hyperglycaemia has been shown to disrupt glucose transport in lymphocytes, which impairs their proliferation capacity, although these changes are partially reversed after insulin treatment in T2DM [73,74]. Moreover, DM is associated with dysregulated apoptosis, resulting in a reduced circulating lymphocyte count in individuals with DM [75]. An elevated neutrophil to lymphocyte ratio (NLR) is commonly observed in individuals with T2DM and has been proposed as a predictive marker of DM complications [76,77]. The highest quartile of NLR (>2.32) was associated with a greater prevalence of common carotid artery plaque and positively associated with CVD compared with the lowest quartile (≤1.38) in individuals with T2DM, suggesting that NLR may be used as a predictor of CVD events [77]. Individuals with early-stage diabetic nephropathy (DN) had significantly higher NLR (2.48 ± 0.59) than T2DM without DN and the healthy controls [78]. An NLR higher than five was associated with an increase in major adverse cardiovascular events after 1 year follow up, including all-cause mortality, stroke, and MI in individuals with DM underwent percutaneous coronary interventions [79]. Conversely, a high lymphocyte to monocyte ratio (LMR) (>2.62) has been linked to reduced cardiovascular mortality in individuals with DM [80].

### 5.3. Monocytes

Monocytes make up around 10% of circulating leukocytes [81]. They migrate to inflamed or injured peripheral tissues, where they differentiate into macrophages [82]. Through phagocytosis and cytokines/chemokine secretions, monocytes contribute to inflammatory status [82]. Monocytosis, or an elevated monocyte count in the blood, is associated with an increased risk of DM and enhanced monocyte recruitment to lesions, promoting atherosclerosis [83,84]. Upon adhesion to the endothelium, monocytes migrate to subendothelial space and attach to a basement membrane protein such as laminin, which facilitates migration and proliferation [85]. Monocytes from individuals with DM display elevated ROS production, enhanced laminin oxidization and increased adhesion to laminin compared to healthy controls, representing an early step in atheroma formation, a common condition in DM [86,87].

Hyperglycaemia upregulates scavenger receptor CD36 expression in macrophages, increasing Ox-LDL uptake and foam cell formation [88,89,90]. The degree of glycaemic control had a different effect on CD36 expression. Controlled DM (HbA1c < 7.0%) as well as poorly controlled DM (>9.4%) show elevated CD36 expression compared to healthy controls by 50% and 130%, respectively [91]. Moreover, poorly controlled DM, identified with HbA1c > 9.4%, was significantly associated with an increase in Ox-LDL uptake by 433% and human aortic endothelial cells attachment by more than 2-fold compared to nondiabetic individuals [91]. Several studies have demonstrated that DM increases monocyte activation, ROS production and proinflammatory cytokine secretions [92,93]. Activated monocytes release monocyte-derived MPs, which are associated with procoagulant activities and cell adhesions [94]. Increased monocyte-derived MP levels in DM correlate with platelet-derived microparticles, platelet activation markers, P-selectin and soluble E-selectin [95,96]. The elevation in monocyte-derived MP level increases the risk of atherosclerosis. Monocyte-derived PM production was significantly increased with diabetic retinopathy and a higher elevation of capillary occlusion [96].

In addition, diabetes-induced metabolic and inflammatory stress alters monocyte biogenesis. The persistence of low-grade inflammatory monocytes has been linked to the reduced expression of interleukin-1 receptor-associated kinase M (IRAK-M), a negative regulator of an innate immune signaling response that suppresses mitogen-activated protein kinase (MAPK) activation and proinflammatory cytokine production [97]. This reduction in IRAK-M expression is driven by the c-Jun N-terminal kinase (JNK)-mediated degradation of the transcription factor small mothers against decapentaplegic homolog 4 (SMAD4), which further enhances JNK activation and sustains inflammatory responses that aggravate atherosclerosis [97].

## 6. Polyphenols

### 6.1. Polyphenols Classification and Metabolism

Polyphenols are a large group of naturally occurring secondary metabolites found in plants, many of them have displayed biological properties such as antioxidant and anti-inflammatory activities [98]. They are classified into four main subgroups based on their chemical structure and the number of phenol rings: (1) flavonoids, characterized by two phenyl rings connected to a heterocyclic ring that are subdivided into flavanols, flavanones, flavones, isoflavones, flavonols, and anthocyanins [98,99], (2) phenolic acids, which are characterized by a single phenolic ring, a carboxylic acid group with one or more hydroxyl group and which include compounds such as caffeic acid and gallic acid [100,101], (3) stilbenes, containing two phenolic rings connected by an ethylene bridge, such as resveratrol [102], and, finally, (4) lignans, composed of two linked phenylpropane (C6–C3) units, such as secoisolariciresinol [103].

Polyphenols undergo a complex process of absorption and metabolism along the gastrointestinal tract, which affects their biological activity [104]. In the stomach, some polyphenols absorb directly, whereas others require further transformation to form active metabolites [105]. In a human trial, anthocyanins have been detected in plasma after 10 min following elderberry extract consumption, which indicates gastric absorption [106]. In the small intestine, some compounds undergo phase I metabolism, including oxidation, reduction and hydrolysis, to facilitate absorption and uptake into enterocytes. Furthermore, they undergo phase II metabolism within the enterocytes and liver, including methylation, glucuronidation, and sulfation, which increases their solubility and enhances bioavailability in the circulation [107]. Polyphenols that are not absorbed in the small intestine, such as daidzein, reach the large intestine, where gut microbiota metabolizes them into secondary metabolites that often exhibit different biological activity compared to the parent compounds [108,109].

The intestinal absorption of polyphenols is dependent on their chemical structure; aglycone forms such as quercetin and phloretin demonstrate more rapid and efficient absorption than their glucosides, which require prior enzymatic hydrolysis before intestinal uptake, as shown in rat intestinal models [110]. Despite these differences between aglycone form and glucoside, the total plasma polyphenol levels were similar after 10 h of ingestion, suggesting that the form administered does not affect the overall bioavailability [111]. However, the elevation in the plasma concentration of polyphenol metabolites varies considerably. For example, phloretin metabolites declined to baseline level after 24 h of oral intake, whereas metabolites from flavonoid such as naringenin remain elevated over same period, likely due to differences in urinary excretion and enterohepatic recycling, which influences metabolites’ half-life [111,112].

### 6.2. Polyphenols and DM

Polyphenols have been widely studied for their beneficial effects on health. A high intake of fruits and vegetables significantly reduced the incidence of T2DM in a Korean adult population; however, no such association was observed in a smaller prospective cohort study [113,114]. A high dietary intake of polyphenols (mean 664 mg/1000 kcal per day) has been generally associated with a reduced incidence of T2DM [17,115]. In a prospective cohort study in Eastern Europe, a higher polyphenol intake, particularly phenolic acids (mean = 1482 mg/day) and flavonoids (mean = 1452 mg/day), was inversely associated with T2DM incidence [116]. Similarly, a large case–cohort study conducted across eight European countries demonstrated that a higher consumption of flavon-3-ols, particularly epigallocatechin gallate (EGCG) (median = 64.48 mg/day) and catechin (median = 27.02 mg/day), as well as flavonols, especially myricetin (median = 5.38 mg/day), was associated with a lower risk of developing T2DM [117].

Several human intervention trials also studied the impact of polyphenols in individuals with DM. A high polyphenol intake (median = 2076 mg/day) has been shown to modulate fasting glucose levels and slightly reduce HbA1c concentrations [16]. In a double blind randomized crossover trial, 600 mg/day of grape seed extract, which is rich in flavonoids, significantly reduced total cholesterol and high-sensitivity CRP after four weeks of supplementation, while increasing whole blood glutathione (GSH), potentially reducing the risk of CVD in individuals with T2DM [118]. Evidence from meta-analysis further indicates that grape seed extract significantly reduced blood pressure and heart rate [119]. Similarly, in another meta-analysis conducted in six randomized control trials showed that resveratrol supplementation enhanced oxidative stress in individuals with T2DM, through increasing GSH and catalase levels, while reducing CRP, lipid peroxide and the oxidative stress score [120]. Moreover, both a single intake of 13.5 g high polyphenol chocolate, as well as acute and chronic (30 days) consumption of a cocoa drink containing 963 mg flavonols per day improved endothelial function, as evidenced by enhanced flow-mediated dilation and reduced adhesion molecules in individuals with T2DM [121,122]. Pycnogenol supplementation (125 mg/day), which is a plant extract derived from pine bark, rich in flavonoid and phenolic compounds, has also been shown to reduce blood pressure and improve endothelial function in individuals with T2DM and hypertension [123]. Following 12 weeks of intervention, the plasma level of endothelin-1 decreased, blood pressure was effectively controlled in approximately two-thirds of intervention group, and the antihypertensive medication doses were reduced by 50% [123]. Significant reductions in LDL cholesterol, HbA1c and fasting blood glucose were also reported [123].

The proposed mechanisms underlying these vascular and metabolic benefits are largely attributed to the antioxidant properties of polyphenols, which may impact antioxidant signaling [122,124]. In addition, polyphenols modulate inflammatory signaling pathways and cellular interaction, thereby contributing to modulated thromboinflammatory risk in DM [125].

### 6.3. The Effect of Polyphenols on Oxidative Stress

**Figure 2:**
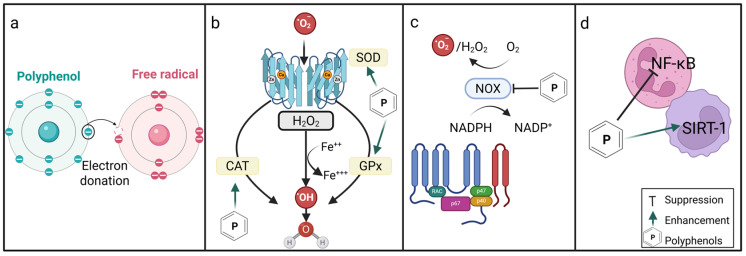
The antioxidant properties of polyphenols and their effect on leukocyte functions. (**a**) Directly scavenged ROS produced by leukocytes. (**b**) Enhanced endogenous antioxidant enzymes formation. (**c**) Reduced ROS production by inhibited NADPH oxidase complexes. (**d**) Upregulated SIRT-1 expression and downregulated NF-kB, which enhances cellular antioxidant defense and reduces ROS levels. Created in BioRender. Muharib, D. (2026) https://BioRender.com/crhjzn7 (accessed on 3 February 2026).

ROS are critically implicated in vascular dysfunction in DM, and accumulating evidence indicates that polyphenols may modulate ROS production. Figure 2 provides a visual representation of the mechanisms by which the antioxidant properties of polyphenols may affect leukocytes. Evidence from an ex vivo study using neutrophils from healthy donors showed that polyphenols such as resveratrol and tannic acid effectively scavenged ROS and hydrogen peroxide (H_2_O_2_) and increased GSH concentration [126,127]. In vitro experiments in human monocytic THP-1 cells under hyperglycaemic conditions (25 mM glucose) showed increased superoxide (O_2_^−^) production and decreased silent information regulator 2 homolog-1 (SIRT-1) expression, nicotinamide adenine dinucleotide (NAD)-dependent deacetylases that regulate cellular metabolism and oxidative stress [128,129]. These effects are consistent with observations in blood samples from individuals with T1DM [129]. These alterations were significantly modulated by resveratrol at doses of 3 and 6 µM which restored SIRT-1 levels and increased forkhead box protein O3a (FOXO3a) expression, a transcription factor that regulates antioxidant gene expression by enhancing the transcription of ROS scavenging enzymes superoxide dismutase 2 (SOD2) and catalase [129]. This suggests that resveratrol mitigates oxidative stress through the activation of SIRT-1 and FOXO3a pathway, which enhances cellular antioxidant defenses and reduces ROS levels [129]. In support of these mechanistic findings, an in vivo trial in individuals with T2DM who were supplemented with resveratrol (placebo; 40 mg or 500 mg/day) for six months demonstrated significantly enhanced SIRT-1 activity and total antioxidant status in peripheral blood mononuclear cells [13]. Furthermore, a low resveratrol dose at 5 µM was sufficient to reduce ROS levels and enhance glutathione peroxidase (GPx) in leukocytes isolated from healthy controls and individuals with Alzheimer’s disease [130]. However, in this model, the antioxidant activity of resveratrol was independent from the SIRT-1 pathway, as it was inhibited by sirtinol [130]. Quercetin, a member of the flavonol subgroup of polyphenols, has also demonstrated significant antioxidant properties [131]. In murine RAW 264.7 macrophages, quercetin significantly reduced ROS production and showed direct O_2_^−^ scavenging activity at doses ranging from 30 to 100 μM [131]. These antioxidant effects were accompanied by the suppression of NF-κB expression and the inhibition of NADPH oxidase complex [131]. Similarly, treatment with quercetin at 20 and 40 µM significantly reduced O_2_^−^ production in stimulated human neutrophils [132,133]. These findings indicate that the antioxidant properties of polyphenols are mediated through different mechanisms involving both direct ROS scavenging and indirect pathways, such as through enhancing endogenous antioxidant enzymes and signaling pathways. However, their efficacy appears to be influenced by doses and underlying health/disease status. Table 1 provides a summary of studies reporting the effects of polyphenols on leukocyte oxidative stress.

### 6.4. The Effect of Polyphenols on NET Formation

**Figure 3:**
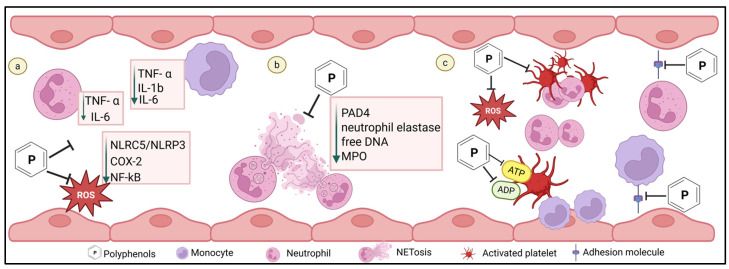
Polyphenol effects on inflammatory markers, NETosis and leukocyte–cellular interaction. (**a**) Polyphenols decrease proinflammatory cytokines in leukocytes through downregulated cellular pathways (NLRC5/NLRP3, COX-2, and NF-kB). (**b**) Polyphenols modulate NETosis. (**c**) Polyphenols modulate leukocyte interactions with other cells, via decreased ROS, reduced platelet activation which decreases neutrophil chemotactic responses, and reduced adhesive molecules. Created in BioRender. Muharib, D. (2026) https://BioRender.com/hkm38cl (accessed on 3 February 2026).

In addition to its central role in oxidative stress, excessive ROS generation acts as a trigger of NET formation, which is strongly linked to thromboinflammatory complications in DM and potentially modulated by polyphenols [42]. Figure 3 provides a visual representation of the mechanisms by which polyphenols may modulate NET formation in leukocytes. Resveratrol, at a dose of 50 µM, significantly suppressed NET release in neutrophils from healthy donors stimulated with biological and non-biological stimuli [127]. This inhibitory effect was independent of the A2A and A2B adenosine receptor activation as well as protein kinase A (PKA) signaling [127]. However, resveratrol effectively reduced H_2_O_2_ levels which regulate NETosis by promoting the release of elastase from azurophilic granules to the nucleus, therefore facilitating chromatin decondensation. Consistent with this mechanism, MPO enzymatic activity and nuclear elastase localization were significantly reduced with resveratrol, suggesting that H_2_O_2_ may be related to the reduction in NETs formation [127]. Furthermore, resveratrol at 100 µM effectively reduced NETosis and the release of free DNA in neutrophils isolated from COVID-19 patients [134]. An in vitro trial demonstrated that resveratrol at 5 µM inhibited NET formation in HL-60 cells by reducing PAD4, neutrophil elastase and MPO expressions [135], while in an in vivo trial the administration of resveratrol intraperitoneally at 15 or 25 mg/kg for two weeks in an arthritis mouse model significantly reduced NETosis, attenuated the increase in PAD4 and cyclooxygenase-2 (COX-2) expression and decreased inflammatory cytokines (TNF-α and IL-1β) with the high dose [136]. Thus, polyphenols have been shown to aid in the suppression of NETosis across diverse diseases, through dose-dependent modulation of ROS, MPO activity, PAD4 and COX-2 expression, highlighting its potential modulatory role in NET-mediated thromboinflammatory complications. Table 2 provides a summary of studies reporting the effects of polyphenols on NET formation.

### 6.5. The Effect of Polyphenols on Inflammatory Markers

Polyphenols have been shown to modulate proinflammatory markers, which contribute to the pathogenesis of thromboinflammation in DM [137]. Figure 3 provides a visual representation of the mechanisms by which anti-inflammatory properties of polyphenols may affect leukocytes. In vitro studies using stimulated neutrophils from healthy donors demonstrated that quercetin reduced cytokine production in a dose-dependent manner, with 40 µM significantly reducing interleukin-6 (IL-6) and 25 µM decreasing TNF-α levels [138,139]. In murine RAW264.7 macrophages, quercetin (10 µM) significantly reduced both the mRNA and protein levels of TNF-α, as well as decreasing inducible nitric oxide synthase (iNOS), IL-1β and IL-6 mRNA levels [140]. These anti-inflammatory effects were associated with the modulation of the nuclear factor erythroid 2-related factor 2 (Nrf2) and NF-kB signaling pathways [140]. Similar anti-inflammatory effects of quercetin have been observed in the human THP-1 monocytic cells line under hyperglycaemic conditions (15 mmol/L) [137]. Quercetin, along with other studied flavonoids, EGCG and rutin, at a dose of 20 µM, significantly attenuated the effect of high glucose on TNF-α and IL-1b levels [137]. Additionally, these flavonoids suppressed COX-2 and NF-kB protein expression, indicating a downregulation of inflammatory pathways [137]. Hyperglycaemia (30 mM glucose) in these cells increased TNF-α, IL-6, and IL-1β levels, in addition to the upregulation of M1 inflammatory factors (CD86 and iNOS), the downregulation of M2 inflammatory factors (CD206 and Arg-1), and the activation of NLRC5/NLRP3 inflammasome pathway [141]. Treatment with quercetin at 16 µM significantly reduced proinflammatory cytokine levels, downregulated iNOS, NLRC5, and NLRP3 protein expression, and upregulated Arg-1 expression. These findings suggest that quercetin effectively attenuated inflammation induced by high glucose and enhanced macrophage polarization from M1 to M2, potentially via the suppression of the NLRC5/NLRP3 pathway [141]. In addition to in vitro evidence, human trials have evaluated the effect of polyphenols on individuals with DM. Resveratrol supplementation (200 mg/day) for 24 weeks significantly reduced serum levels of TNF-α, CRP and IL-6 compared to baseline levels in individuals with T2DM [142]. However, the findings in vivo are inconsistent. In another randomized trial, resveratrol (480 mg/day) for 4 weeks reduced IL-6 levels but had no effect on TNF-α in T2DM [143]. Furthermore, a higher resveratrol dose administered over a longer duration (800 mg/day for 8 weeks) for individuals with T2DM improved blood pressure and fasting blood glucose levels but did not significantly alter proinflammatory cytokine levels [144]. These discrepancies highlight the importance of considering compound bioavailability metabolism in study design. Table 3 provides a summary of studies reporting the effects of polyphenols on leukocyte proinflammatory cytokine productions.

### 6.6. The Effect of Polyphenols on Leukocyte Interaction with Other Cells

Leukocyte adhesion to the endothelium and the formation of leukocyte–platelet aggregates may contribute to thromboinflammatory status and increase thrombosis risk in DM [10,145]. Several studies suggest that polyphenols can attenuate these interactions by reducing the expression of adhesin molecules and modulating leukocyte–platelet aggregation [146,147]. Figure 3 provides a visual representation of polyphenol mechanisms that may affect leukocyte–cell interactions. For example, both resveratrol and quercetin at 1 fM to 100 μM have been shown to reduce platelet activation through lowering adenosine diphosphate (ADP) and adenosine triphosphate (ATP) levels via the downregulation of JNK and MAPK pathways and reduced chemotactic responses in neutrophils from healthy donors, indicating their inhibitory role in platelet–neutrophil interactions [147]. The treatment of neutrophils from healthy donors with quercetin at 40 µM reduced neutrophil adhesion molecules expression CD11b and CD18, which might reduce neutrophil adhesion to the endothelium [132]. However, another study that tested the effect of quercetin at 10 µM, and reported no effect on CD11b expression [148]. In vivo, the intraperitoneal administration of resveratrol (20 mg/kg/day) to hypercholesteremic rats significantly reduced platelet–neutrophil formation and ROS levels, with aggregate reduction that was positively correlated with ROS inhibition [146]. Furthermore, in an intestinal–endothelial–macrophages coculture, quercetin at 10 µM significantly inhibited monocyte–endothelial interactions by reducing the adhesion of monocytes (THP-1 cells) to endothelium and lowering endothelial secretion of soluble vascular cells adhesion molecules-1 (sVCAM-1), demonstrating its ability to interfere with monocyte–endothelial aggregation [149]. These studies suggest that polyphenols can attenuate leukocyte–cell interactions; however, the variability in effective concentrations, cell models used and experimental conditions result in considerable differences in the reported effects. Table 4 provides a summary of studies reporting the effects of polyphenols on leukocyte- cell interactions.

## 7. Conclusions

The global prevalence of DM is rising, with cardiovascular complications being the major risk associated with DM. Polyphenols have demonstrated a range of properties such as antioxidant and anti-inflammatory activity. This review discussed the potential protective role of polyphenols in modulating leukocyte functions related to thromboinflammatory status in DM, particularly through decreasing oxidative stress, attenuating inflammatory markers, and modulating leukocyte bioenergetics and function. However, the current research in this area is limited due to the fact that most studies are in vitro, with heterogeneous intervention durations, polyphenol types, doses, and classes, and that only small clinical trials directly evaluate thromboinflammatory mechanisms induced by leukocytes in individuals with DM. Therefore, further research is required to investigate the effect of polyphenols on leukocyte activation, interaction, and aggregation that drive thrombosis. In addition, studies are needed to clarify how bioavailability and metabolism influence the capacity of polyphenols to modulate thromboinflammation in DM, define optimal dosages, determine potential harmful effects of high or prolonged exposures, and assess their use as adjunctive therapy in DM.

## Figures and Tables

**Table 1:** Polyphenol interventions and their observed effects with potential to modulate leukocyte oxidative stress.

Polyphenol Class	Polyphenol Type	Leukocyte Type	Modulated Function
Tannins and stilbenes	Tannic acid and resveratrol [126] Dose: 1 to 100 μM	Neutrophils from healthy donors Stimulated with 12-O-tetradecanoyl-phorbol-13-acetate (TPA)	↓ ROS and H_2_O_2_ levels (50 μM) ↑ GSH levels (100 μM)
Stilbenes	Resveratrol [127] Dose: 50 μM	Neutrophils from healthy donors Stimulated with PMA or lipopolysaccharide (LPS)	↓ ROS and H_2_O_2_ levels
	Resveratrol [129] Dose: 3 or 6 μM	THP-1 cells in normoglycaemic (5.5 mmol/L) or hyperglycaemic (25 mmol/L)	↓ O_2_^−^ production and p47 phox expression ↑ SIRT-1 activity FOXO3a expression
	Resveratrol [13] Dose: 40 mg or 500 mg, orally, for 6 months	PBMCs from 128 individuals with T2DM	↑ SIRT-1 levels and total antioxidant status
	Resveratrol [130] Dose: 5 μM	Leukocytes from individuals Alzheimer	↓ ROS levels independently from SIRT-1 pathway ↑ GPx levels
Flavonoid (flavonol)	Quercetin [131] Dose: 1–100 μM	Murine macrophage RAW264.7 cells Stimulated with PMA or zymosan	↓ O_2_^−^ production (30 μM) ↓ NF-κB expression (100 μM)
	Quercetin [132] Dose: 40 μM	Neutrophils from healthy donors Stimulated with LPS	↓ O_2_^−^ production
	Quercetin [133] Dose: 20 μM	Neutrophils from healthy donors Stimulated with y N-formyl-methionyl-leucyl-phenyl alanine (fMLP).	↓ O_2_^−^ production through suppression of tyrosyl phosphorylated proteins

↓ indicates significant decrease, ↑ indicates significant increase.

**Table 2:** Polyphenol interventions and their observed effects with potential to modulate NET formation.

Polyphenol Class	Polyphenol Type	Leukocyte Type	Modulated Function
Stilbenes	Resveratrol [127] Dose: 50 μM	Neutrophils from healthy donors. Stimulated with PMA or LPS	↓ NETs formation and H_2_O_2_ levels. Inhibiting MPO activity and modulating neutrophil elastase localization.
	Resveratrol [134] Dose: 100 μM	Neutrophils from COVID-19 patients. Stimulated with PMA	↓ NET formation and free DNA
	Resveratrol [135] Dose: 5 μM	HL-60 cells. Stimulated with LPS.	↓ NET formation, PAD4, neutrophil elastase and MPO expressions
	Resveratrol [136] Dose: 5 or 25 mg/kg, intraperitoneally for 2 weeks	Male wild type C57BL/6 mice (arthritis model)	↓ NETs release ↓ PAD4 and COX-2 expressions

↓ indicates significant decrease.

**Table 3:** Polyphenol interventions and their observed effects with potential to modulate leukocyte proinflammatory cytokine productions.

Polyphenol Class	Polyphenol Type	Leukocyte Type	Modulated Function
Flavonoid (flavonol)	Quercetin [138] Dose: 40 μM	Neutrophils from healthy donors. Stimulated with LPS	↓ IL-6 secretions and mRNA expressions
Flavonoid (flavonol and flavone)	Quercetin and vitexin [139] Dose: 25 μM	Neutrophils from healthy donors. Stimulated with PMA	↓ TNF-α concentrations and MPO activity
Flavonoid (flavonol and flavanol)	Quercetin, rutin and EGCG [137] Dose: 20 μM	THP-1 cells in normoglycaemic (5.5 mmol/L) or hyperglycaemic (15 mmol/L)	↓ TNF-a, IL-1β, COX-2 and NF-kB expressions
Flavonoid (flavonol)	Quercetin [140] Dose: 10 μM	Murine macrophage RAW264.7 cells Stimulated with LPS	↓ TNF-α mRNA and secretion ↓ IL-β, IL-6, iNOS mRNA and protein levels ↓ NF-κB transactivation ↑ Nrf2 transactivation
	Quercetin [141] Dose: 16 μM	Murine macrophage RAW264.7 cells with glucose concentrations (5 mM, 10 mM, 20 mM, or 30 mM)	↓ TNF-α, IL-1β, and IL-6 concentrations ↓ NLRC5, and NLRP3 protein levels
Stilbenes	Resveratrol [142] Dose: 200 mg/day, orally, for 24 weeks	Serum from 110 individuals with T2DM	↓ TNF-α, CRP and IL-6 levels
	Resveratrol [143] Dose: 480 mg/day, orally, for 4 weeks	Serum from 43 individuals with T2DM	↓ IL-6 levels, but no effect on TNF-α
	Resveratrol [144] Dose: 800 mg/day, orally, for 8 weeks	Plasma and PBMCs from 45 individuals with T2DM	No effect on proinflammatory cytokine levels

↓ indicates significant decrease, ↑ indicates significant increase.

**Table 4:** Polyphenol interventions and their observed effects with potential to modulate leukocyte-cell interactions.

Polyphenol Class	Polyphenol Type	Leukocyte Type	Modulated Function
Stilbenes and flavonoid (flavonol)	Resveratrol and quercetin [147] Dose: 10 fM to 100 μM	Neutrophils from healthy donors with thrombin activated platelets	↓ ATP and ADP release from platelets ↓ Chemotactic response of neutrophils to activated platelets ↓ Thrombin signaling by suppressing MAPK and JNK activities
Flavonoid (flavonol)	Quercetin [132] Dose: 40 μM	Neutrophils from healthy donors. Stimulated with LPS	↓ Adhesion molecule expression (CD11b and CD18)
	Quercetin [148] Dose: 10 μM	Neutrophils from healthy donors. Stimulated with LPS or fMLP	No effect on neutrophil adhesion molecule expressions
Stilbenes	Resveratrol [146] Dose: 20 mg/kg/day, intraperitoneally for 20 days	Hypercholesteremic rats	The reduction in ROS levels by resveratrol was positively correlated platelet-neutrophil aggregation.
Flavonoid (flavonol)	Quercetin [149] Dose: 10 μM	THP-1 cells	↓ Monocyte adherence to a in LPS-stimulated endothelial cell line ↓ IL-6 and sVCAM-1 secretion

↓ indicates significant decrease.

## Data Availability

No new data were created or analyzed in this study. Data sharing is not applicable to this article.

## Abbreviations

The following abbreviations are used in this manuscript:

ADP	Adenosine diphosphate
AGEs	Advanced glycation end products
APTT	Activated partial thromboplastin time
ATP	Adenosine triphosphate
CAD	Coronary artery disease
COX-2	Cyclooxygenase-2
CRP	C-reactive protein
CVD	Cardiovascular diseases
dsDNA	Double-stranded DNA
DM	Diabetes mellitus
DN	Diabetic nephropathy
DNA	Deoxyribonucleic acid
EGCG	Epigallocatechin gallate
FOXO3a	Forkhead box protein O3a
GSH	Glutathione
GSSG	Oxidized glutathione
H_2_O_2_	Hydrogen peroxide
HbA1c	Hemoglobin A1c
IL	Interleukin
iNOS	Inducible nitric oxide synthase
IRAK-M	Interleukin-1 receptor-associated kinase M
JNK	c-Jun N-terminal kinase
LMR	Lymphocyte to monocyte ratio
LPS	Lipopolysaccharide
MAPK	Mitogen- activated protein kinase
MI	Myocardial infarction
MPO	Myeloperoxidase
MPs	Microparticles
NAD	Nicotinamide adenine dinucleotide
NADPH	Nicotinamide adenine dinucleotide phosphate
NETs	Neutrophil extracellular traps
NF-κB	Nuclear factor–κB
NLR	Neutrophil to lymphocyte ratio
Nrf2	Nuclear factor erythroid 2-related factor 2
O_2_^−^	Superoxide
PAD4	Peptidyl arginine deiminase 4
PBMCs	Peripheral blood mononuclear cells
PKA	Protein kinase A
PMA	Phorbol 12-myristate 13-acetate
ROS	Reactive oxygen species
SIRT-1	Silent information regulator 2 homolog-1
SMAD4	Small mothers against decapentaplegic homolog 4
SOD2	Superoxide dismutase 2
sVCAM-1	Soluble vascular cells adhesion molecules-1
T1DM	Type 1 diabetes mellitus
T2DM	Type 2 diabetes mellitus
TF	Tissue factor
TNF-α	Tumor necrosis factor-α
TPA	12-O-tetradecanoyl-phorbol-13-acetate
